# Predictors of COVID-19 Vaccine Hesitancy in South African Local Communities: The VaxScenes Study

**DOI:** 10.3390/vaccines10030353

**Published:** 2022-02-25

**Authors:** Patrick D. M. C. Katoto, Saahier Parker, Nancy Coulson, Nirvana Pillay, Sara Cooper, Anelisa Jaca, Edison Mavundza, Gregory Houston, Candice Groenewald, Zaynab Essack, Jane Simmonds, Londiwe Deborah Shandu, Marilyn Couch, Nonkululeko Khuzwayo, Nobukhosi Ncube, Phelele Bhengu, Heidi van Rooyen, Charles Shey Wiysonge

**Affiliations:** 1 Cochrane South Africa, South African Medical Research Council, Cape Town 7501, South Africa; sara.cooper@mrc.ac.za (S.C.); anelisa.jaca@mrc.ac.za (A.J.); edison.mavundza@mrc.ac.za (E.M.); phelelebhengu@googlemail.com (P.B.); charles.wiysonge@mrc.ac.za (C.S.W.); 2 Department of Global Health, Faculty of Medicine and Health Sciences, Stellenbosch University, Cape Town 7505, South Africa; 3 Centre for General Medicine and Global Health, Department of Medicine, University of Cape Town, Cape Town 7505, South Africa; 4 Human Sciences Research Council, Cape Town 8000, South Africa; sparker@hsrc.ac.za (S.P.); ghouston@hsrc.ac.za (G.H.); cgroenewald@hsrc.ac.za (C.G.); zessack@hsrc.ac.za (Z.E.); ldshandu@hsrc.ac.za (L.D.S.); marilyncouch12@gmail.com (M.C.); nkhuzwayo@hsrc.ac.za (N.K.); hvanrooyen@hsrc.ac.za (H.v.R.); 5 The Sarraounia Public Health Trust, Johannesburg 2193, South Africa; nancy@sarraounia.org or nirvana@sarraounia.org or nobukhosi2303@gmail.com (N.N.); 6 Wits Mining Institute, University of the Witwatersrand, Johannesburg 2050, South Africa; 7 School of Public Health, University of the Witwatersrand, Johannesburg 2050, South Africa; 8 Department of Psychology, Rhodes University, Grahamstown 6140, South Africa; 9 South African Research Ethics Training Initiative (SARETI), University of KwaZulu-Natal, Pietermaritzburg 3209, South Africa; 10 South African Medical Research Council, Cape Town 7501, South Africa; jane.simmonds@mrc.ac.za; 11 Department of Medicine, Faculty of Health Sciences, University of Cape Town, Cape Town 7935, South Africa; 12 SAMRC-Wits Developmental Pathways for Health Research Unit, Faculty of Health Sciences, University of the Witwatersrand, Johannesburg 2050, South Africa; 13 School of Public Health and Family Medicine, University of Cape Town, Cape Town 7935, South Africa

**Keywords:** immunization, vaccine acceptance, behaviors, social drivers, SARS-CoV-2

## Abstract

South Africa launched a mass COVID-19 vaccination campaign in May 2021, targeting 40 million adults. Understanding predictors of COVID-19 vaccine intentions was required to achieve this goal. We conducted a population-based survey in June–July 2021 using the WHO Behavioral and Social Drivers (BeSD) of COVID-19 Vaccination tool to determine predictors of vaccine hesitancy, defined as intention to refuse or uncertainty whether to accept COVID-19 vaccination. There were 1193 participants, mean age 39 (standard deviation 15) years, and 53% women, of whom 58% trusted information provided by healthcare workers and 32% were vaccine hesitant. Independent predictors of vaccine hesitancy included concerns about side effects (odds ratio (OR) 11.41; 95% confidence interval (CI) 3.5–50.80), lack of access to the online vaccine registration platform (OR 4.75; CI 2.15–10.37), distrust of government (OR 3.0; CI 1.33–6.77), belief in conspiracy theories (OR 3.01; CI 1.32–6.77), having no monthly income (OR 1.84; CI 1.12–3.07), and depending on someone else to make vaccination decision (OR 2.47; CI 1.06–5.77). We identified modifiable predictors of vaccine hesitancy at the start of South Africa’s COVID-19 vaccination rollout. These factors should be addressed by different stakeholders involved in the national immunization program through tailored communication and other effective strategies that increase vaccine literacy, reach low-income households, and engender confidence in government.

## 1. Introduction

South Africa has reported the highest number of cases of COVID-19 in Africa despite implementing various control measures [1]. The country responded to the COVID-19 pandemic by imposing an extended period of nationwide lockdown beginning on 26 March 2020 [2]. In addition to the lockdown, the country’s pandemic response included implementing infection prevention and control measures such as frequent handwashing, use of alcohol-based hand sanitizers, and cough hygiene [3]. The government also imposed mandatory use of various preventive non-pharmaceutical interventions in public (including face masks and physical distancing) as well as containment measures (such as case identification, isolation, contact tracing, and quarantine) that have played an important role in decreasing transmission [4,5]. The hope for the return to normalcy has been renewed with the development and approval for emergency use of several vaccine candidates against SARS-CoV-2 to complement the non-pharmaceutical interventions [6,7]. Current evidence suggests that vaccines are among the most effective prevention tools available to address the unprecedented global health crisis as they prevent infection transmission and reduce hospitalization and death [8]. Thus, efforts to control the COVID-19 pandemic largely depend on the development, acquisition, and administration at large scale of effective vaccines.

The South African government secured enough COVID-19 vaccines to vaccinate at least 40 million people by December 2021 [9]. The safety and efficacy of the vaccines used in the national vaccine rollout (the Pfizer-BioNTech’s messenger RNA and the Johnson and Johnson’s viral vector vaccines) have been endorsed by the national regulatory authority, the South African Health Products Regulatory Authority (SAPHRA). The country’s vaccine strategy [9] was implemented in three stages, beginning with the most vulnerable such as frontline healthcare workers, then other essential workers, people in congregate settings, people over 60 years old, people over 18 years old with comorbidities, and finally every person over the age of 18. The goal was to vaccinate 67% of the population by the end of 2021. However, the vaccination program often faces challenges such as supply and availability, access, and vaccine hesitancy [10]. As of 23 December 2021, only 38.8% of the adult population was fully vaccinated [11]. Vaccine hesitancy was ranked among the ten greatest threats to global health by the World Health Organization (WHO) in 2019 [12]. Vaccine hesitancy is an old health issue and has been defined as a “delay in acceptance or refusal of vaccines despite availability of vaccination services” [13]. Emerging global evidence suggests that about one in three individuals is more likely to delay or refuse the COVID-19 vaccine [14,15,16,17,18]. In South Africa, Cooper et al. recently summarized surveys and found an acceptance for the vaccine ranging from 52% to 82% [19].

Vaccine hesitancy is complex and context-specific and differs across time, place, and type of vaccines [20]. It has been strongly associated with lack of confidence in the safety and effectiveness of vaccines [21,22]. Moreover, the low perception of disease risk, also termed complacency, can also drive vaccine hesitancy. In the context of a new vaccine such as the COVID-19 vaccine, where there is a significant knowledge gap among political leaders, community, and even health workers [23], uncertainty could increase vaccine hesitancy [24,25]. Consequently, organizational health literacy [26], the extent to which organizations allow people to locate, comprehend, and utilize information and services to inform health-related choices and actions for themselves and others in an equitable manner, might determine vaccine uptake in settings with variable resources.

Contextual factors that shape the enabling environment for vaccine uptake within a middle-income country such as South Africa might be expected to play a bigger role in levels of vaccine hesitancy when compared with the results from high income countries [27]. For example, while concern about vaccine safety and trust in government and scientists are common to diverse settings, the influence of spouses and partners in the decision-making process [28] and vaccine availability and cost of transport are regular barriers to access to health services in resource constrained areas [19,27]. Therefore, we surveyed communities in marginalized areas and considered people living in urban, urban-informal, and semi-rural areas in South Africa to assess factors that encourage or impede vaccine uptake to inform the implementation of mass COVID-19 vaccination programs at the community level.

## 2. Materials and Methods

### 2.1. Study Design, Setting and Population

From 6 June to 30 July 2021, we performed a cross-sectional survey in four communities. This survey was nested within the VaxScenes Study, a ward-based mixed-methods study assessing factors associated with vaccine uptake in South African communities. The four selected communities are described in Table S1. Purposive-convenience sampling was used in the selection of sites. In this regard, some sites were identified as members of the research team had established networks within these communities while other sites were well-known to the study team (government agencies). This familiarity facilitated rapport building with community gatekeepers and access to the communities. The study sites were also purposively selected to include a mix of sites from different provinces seriously affected by COVID-19 which included wards that reflected formal and informal urban contexts, peri-urban and rural environments, as well as diversity of race and income. In summary, wards (geopolitical subdivisions of municipalities used for electoral purposes) were selected in three diverse South African provinces (KwaZulu Natal, Gauteng and the Western Cape provinces) with the highest numbers of COVID-19 cases in South Africa since the beginning of the pandemic [29]. The two wards in KwaZulu Natal were Wentworth (urban) and Sweetwaters (semi-rural). Alexandra, an urban informal community, was selected in Gauteng and Rylands Estate, an urban (middle to upper-middle class) site was selected in the Western Cape.

We used a random stratified sampling strategy with an n = 300 sample size for each study location. The sample proportions were calculated using age and gender data from the 2011 StatsSA census (Table S1). Following that, we constructed a sample with a 10% oversample based on the age and gender distributions within each chosen community/ward. These were used to design sampling frames and to guide data collection to ensure that the sample obtained was representative.

Additionally, we consulted with local key stakeholders in each community to assist in determining the final selection of survey respondents. In instances where specific demographics—by age or gender—were difficult to locate, local key informants/stakeholders were able to facilitate access to individuals meeting such sampling requirements (senior’s clubs or women’s organizations, for example). Local community leaders and service providers assisted with a list of ward-level stakeholders. Finally, the survey’s inclusion criteria required respondents to be 18 years or older at the time of the survey, to live or work in the ward where the survey was conducted.

### 2.2. Questionnaire

The World Health Organization Behavioral Social Drivers of COVID-19 vaccination (BeSD) tool [30] for adult populations was used to assess vaccine hesitancy and associated covariates. BeSD assesses four domains that impact vaccine uptake: what individuals think and feel about vaccinations; societal processes that promote or hinder vaccination; individual motivations (or hesitancy) to seek vaccination; and practical elements involved in obtaining and getting immunization. However, there is limited experience of the application of the BeSD tool in a low- and middle-income country (LMIC) such as South Africa and our study serves as an opportunity to assess its usefulness in the African continent and for other LMICs. The survey tool was extensively adapted for use at the community level in South Africa although researchers still keeping the underlying structure determined by the four domains. Additional sociodemographic information was collected to assist researchers in contextualizing the findings, as well as direct questions about the specifics of accessing a vaccination. The language and tone of the survey tool was also adapted to the South African context and was made sensitive to the historical moment (time) the survey was conducted. For example, at the time of the data collection most adults had not been called for a vaccination and those that had been targeted were required to register on the online government vaccination portal.

The survey tool was in English and was translated into additional languages, isiZulu and Afrikaans, and back translated into English for consistency. From the 25th of May 2021 to the 3rd of June 2021, the BeSD survey tool was piloted at all sites (n = 85), and minor adjustments were made as recommended by the WHO [30]. Data were captured onto tablets during the interview. The survey was conducted by trained researchers working on site.

### 2.3. Data Analysis

We used Statistical Package for Social Sciences (IBM SSPS Inc., Chicago, IL, USA) V.27.0 and R (The R Foundation for Statistical Computing, Vienna, Austria)) V4.0.5 software for data cleaning and analyses. Vaccine hesitancy, the outcome of interest was measured using the question: “When available, would you take the COVID-19 vaccine?”. The answers included “yes definitely”, “yes probably”, “I am uncertain at this stage”, “no probably”, “no definitely” and “I have already taken the vaccine”. To have a binary outcome, we defined vaccine hesitancy as “I am uncertain OR no probably OR no definitely” whilst, “I have already taken the vaccine OR yes definitely OR yes probably” were merged to define vaccine acceptance. Similarly, for some explanatory variables such as, “Do you think most adults you know in this local community will get a COVID-19 vaccine, if government makes it available to them?”, the responses included “yes”, “no” and “not sure”. For this type of question, we merged “no” and “not sure” to become “no”. To describe data, we summarized data as count and percentages for categorical variables and mean (standard deviation (SD)) for continuous variables. Chi-square tests, *t*-test and analyses of variances were used as appropriate for group comparisons. We built three logistic regression models using generalized linear model in Finalfit 1.0.4 package in R to determine predictors strongly associated with hesitancy to the COVID-19 vaccine among respondents. The first model adjusted for sociodemographic variables, the second model adjusted for COVID-19 vaccine-related variables captured by the BeSD tool, and the last model only included predictors significantly associated with vaccine hesitance in models 1 and 2. Both models 2 and 3 were adjusted for age as continuous variables and sex as binary variables regardless of their level of significance. Listwise deletion was used to handle missing data. A *p*-value 0 < 0.05 was used to indicate statistically significant results.

### 2.4. COVID-19 Precautions

The research team adopted an approach to data collection that is compliant with all the rules pertaining to the COVID-19 response. Researchers wore masks, practiced social distancing, and used hand sanitizer in all interactions with respondents. When needed, respondents received PPE including face masks and hand sanitizer and all survey interviews were conducted in outdoors or in well ventilated venues. Some data were collected online.

### 2.5. Ethical Clearance

Every effort was made to ensure that the research process was socially sensitive and respected the rights of all participants. The study was approved by the University of the Witwatersrand Human Research Ethics Committee (Ref: H21/02/05), the South African Medical Research Council (SAMRC) Human Research Ethics Committee (Ref: EC022-5/2021), and the Human Sciences Research Council (HSRC) Research Ethics Committee (Ref: REC 12/04/21). The aim and the objectives of the study were clearly explained to respondents verbally, each respondent was provided a Participant Information Sheet which included referral details for a local social workers and other COVID-19 resources in the community. Informed consent was obtained from each respondent. Participation in this study was entirely voluntary and participants were assured that they could withdraw at any time without any consequences.

## 3. Results

### 3.1. Participant Characteristics and Distribution of COVID-19 Vaccine Hesitancy

Table 1 presents baseline characteristics of study participants and prevalence of COVID-19 vaccine hesitancy across key demographic and behavioral and social drivers (BeSD—thinking and feeling, social processes, motivation, and practical issues). Of the 1193 participants who completed the survey, the majority were Black (50%) and female (53%), aged 39 years (SD:15), with matric or tertiary level of education (65%) but unemployed (51%) and with no monthly income (30.8%).

**Figure 1:**
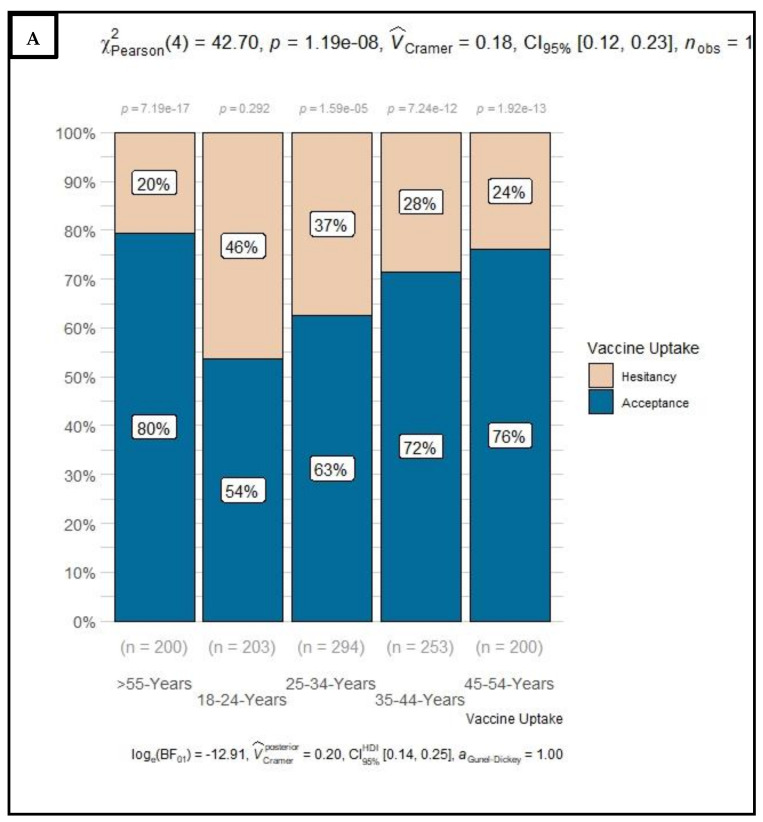

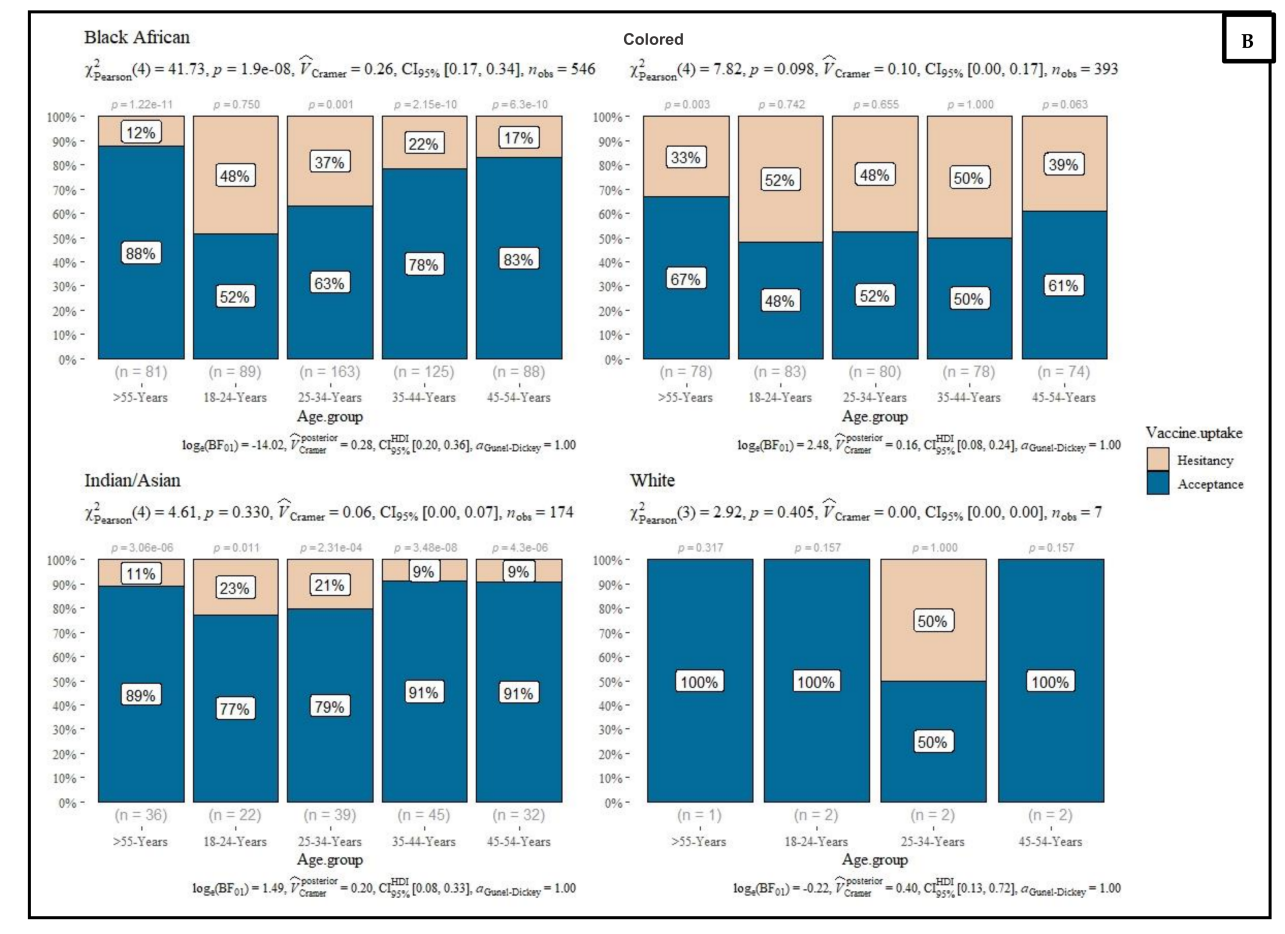

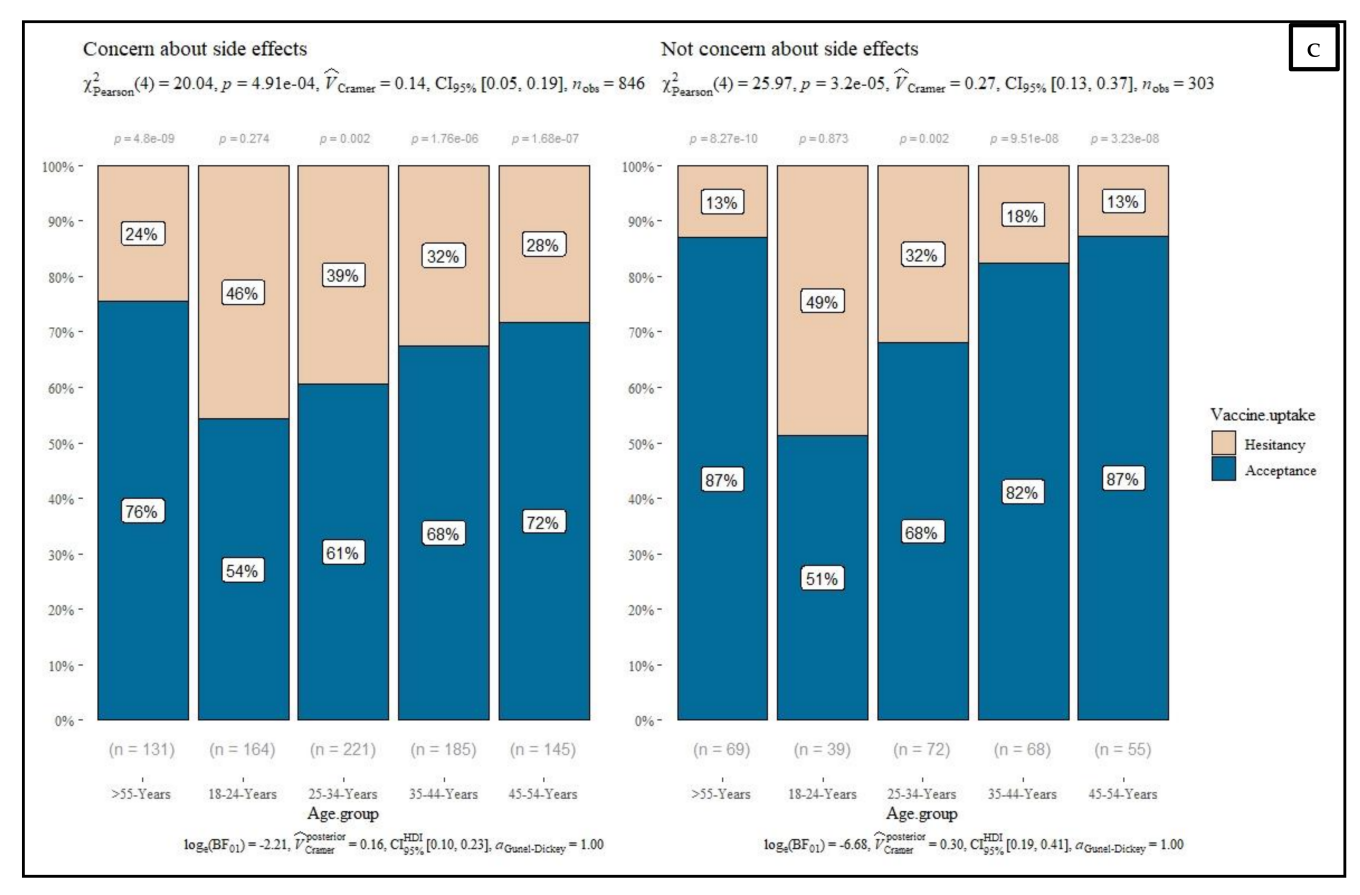

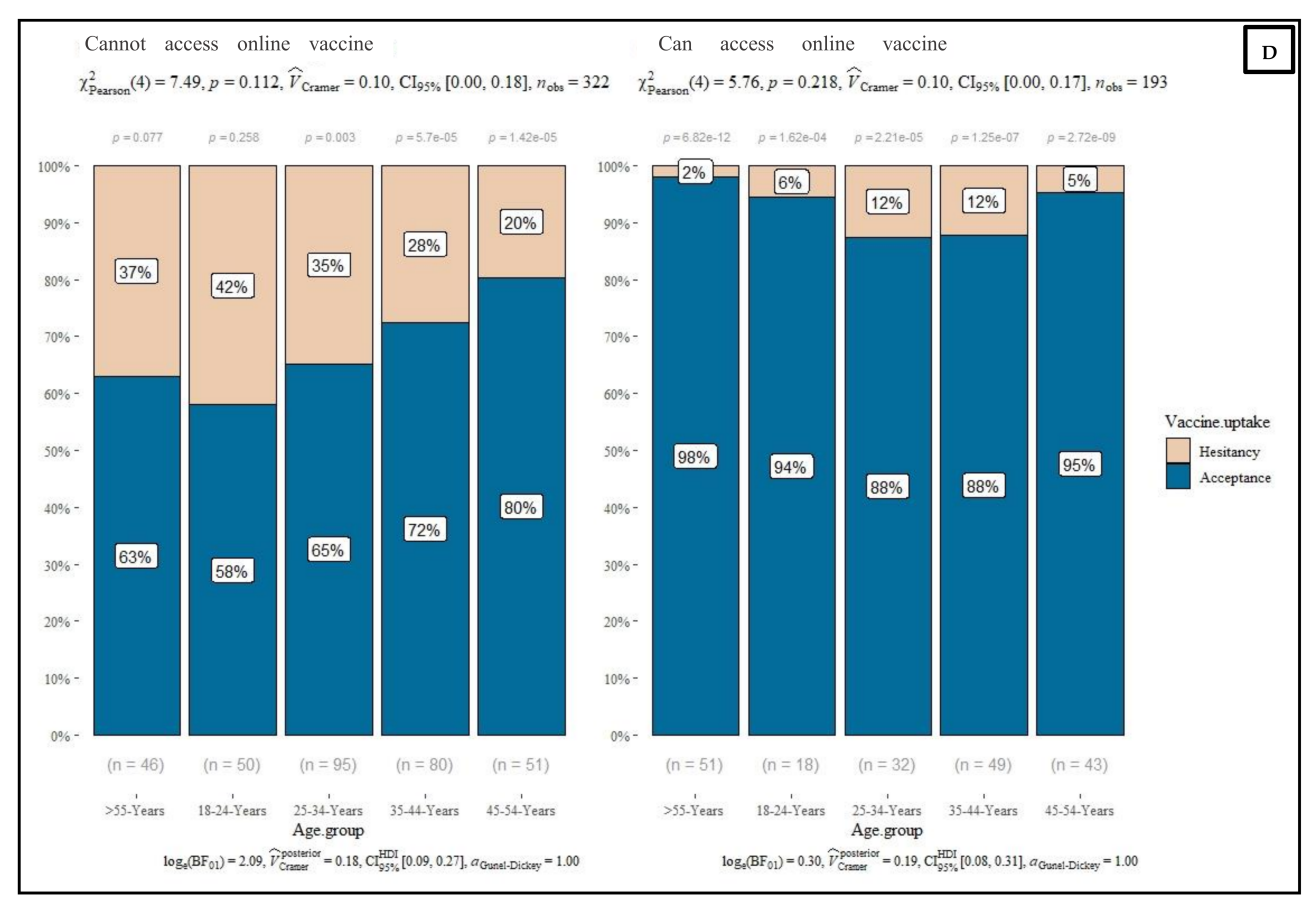

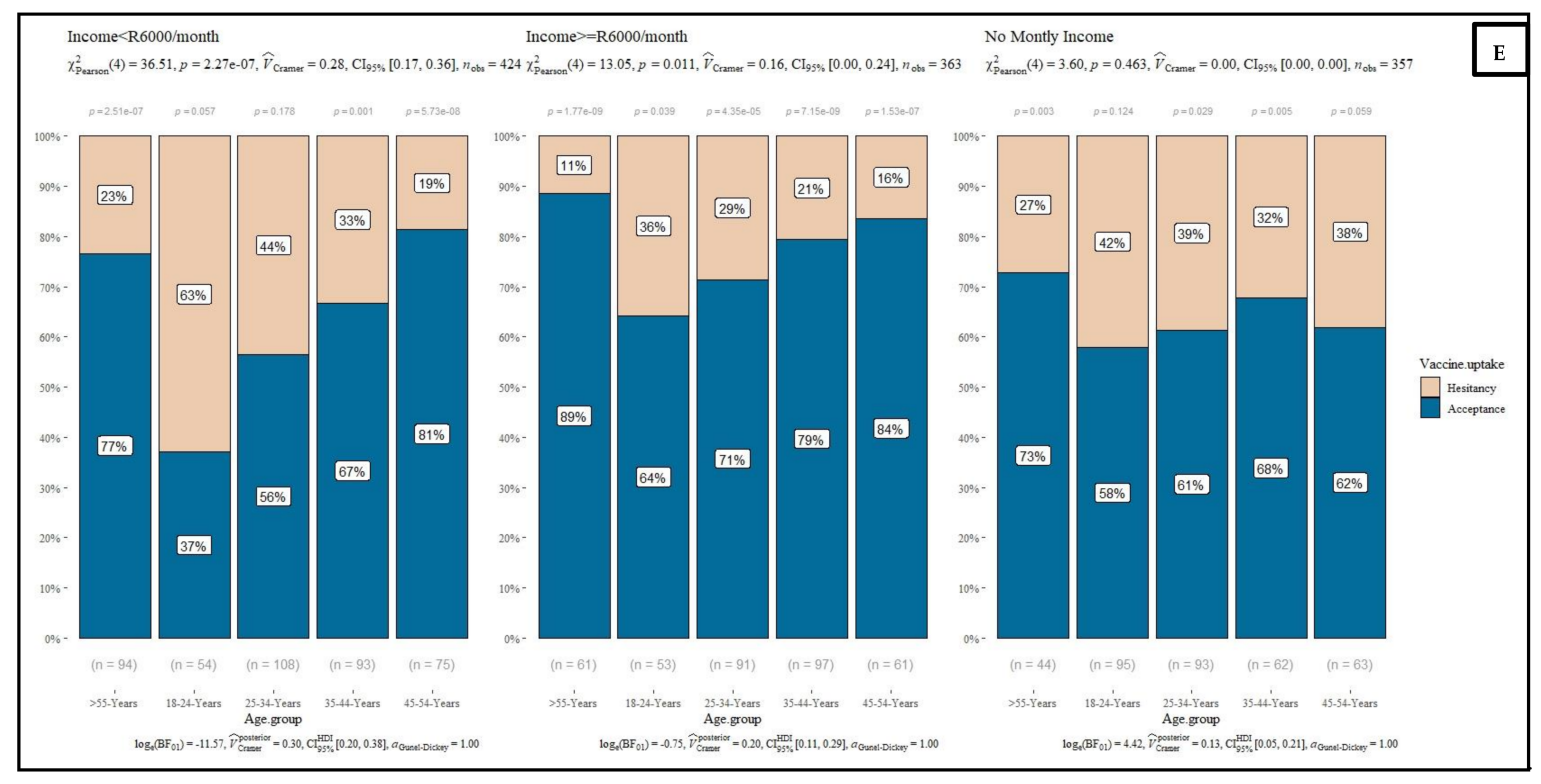
Distribution of COVID-19 vaccine uptake by age groups (**A**) and, by ethnic groups (**B**), by level of concern about side effects (**C**), by ability to access the government online COVID-19 platform (**D**) and by reported monthly income (**E**) among respondents from four local communities in South Africa, from the 6th of June to the 30th 2021.

Overall, 32% of respondents were hesitant to receive the COVID-19 vaccine once available: more than half (56%) were young adults between 18–34 years, Colored (49%) or Black (45%), unemployed (58%) and with low (52%) or no (20%) monthly income. While female (52%) respondents and those with high level of education (64%) showed a high level of hesitancy, but the difference did not reach the statistical level of significance. Sensitivity analyses by age groups (Figure 1 and Figure S1) revealed that among respondents at very high risk of dying with COVID-19 infection (>55 years), 20% were hesitant to receive COVID-19 vaccine once available with no distinction related to education attainment (22% and 19% among those with no or some schooling and those with matric or tertiary education, respectively). However, this proportion was higher in Alexandra (33%) and Wentworth (37%) wards/counties and among Colored (33%), females (25%), and those with low (23%) or no monthly income at all (27%) and who were concerned about side effects (24%) and who could not get access to the government’s online COVID-19 platform.

Most respondents believed that people who do not get the vaccine are taking risks both for their own health and that of the wider community (66%), that most adults in their local community will get a COVID-19 vaccine if the government makes it available to them (73%), and that family/friends (60%), local wards councilors (61%), religious leaders (57%), political leaders (60%), and traditional leaders (32%) would want them to get a COVID-19 vaccine. Compared to other places such as workplace or community centers/meeting halls/local shopping centers, more than half of respondents indicated that they would prefer to be vaccinated in a health-related facility such as a hospital, clinic, or pharmacy. Equally, 58% of participants considered healthcare workers (nurse/doctor/community health workers) as a reliable source to access COVID-19 related information (Table S2 and Table 2).

On a scale of 1 (not at all) to 4 (very much), more than half (55%) of respondents indicated moderate to high levels of trust in the national government to manage the rollout of the COVID-19 vaccine. Moderate to high levels of trust were also observed towards the pharmaceutical industry’s development of COVID-19 vaccines (62%) and scientists advising the national government about COVID-19 vaccines (67%). Among actions needed to facilitate uptake of the COVID-19 vaccine at the community level, the most preferred strategies were ensuring vaccines were “free of charge” (74%) and available to all adults (74%), as well as providing more education about COVID-19 (69%) and involving religious and community leaders in COVID-19 education programs (67%) (Table S2).

### 3.2. Factors Associated with COVID-19 Vaccine Hesitancy

**Figure 2:**
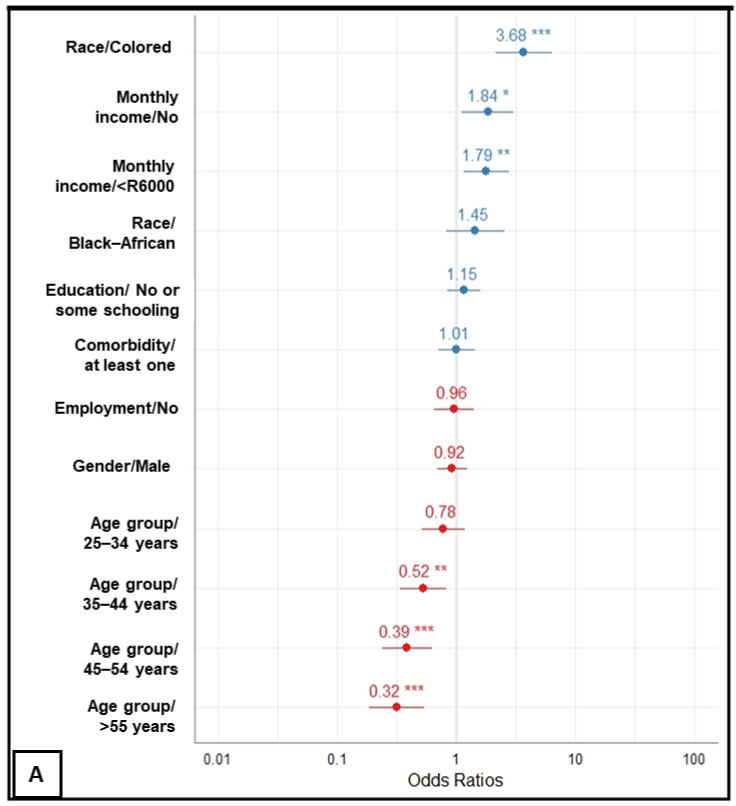

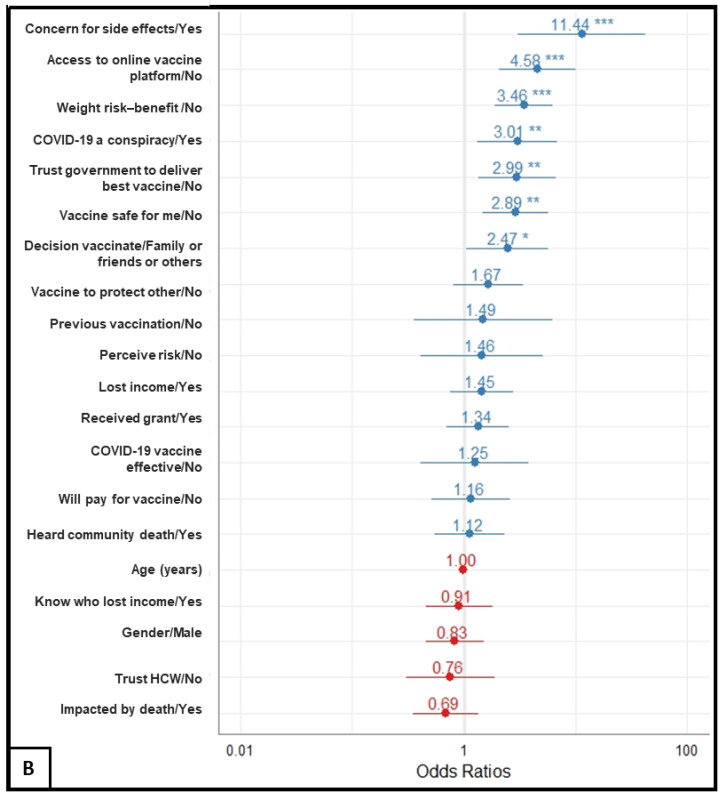

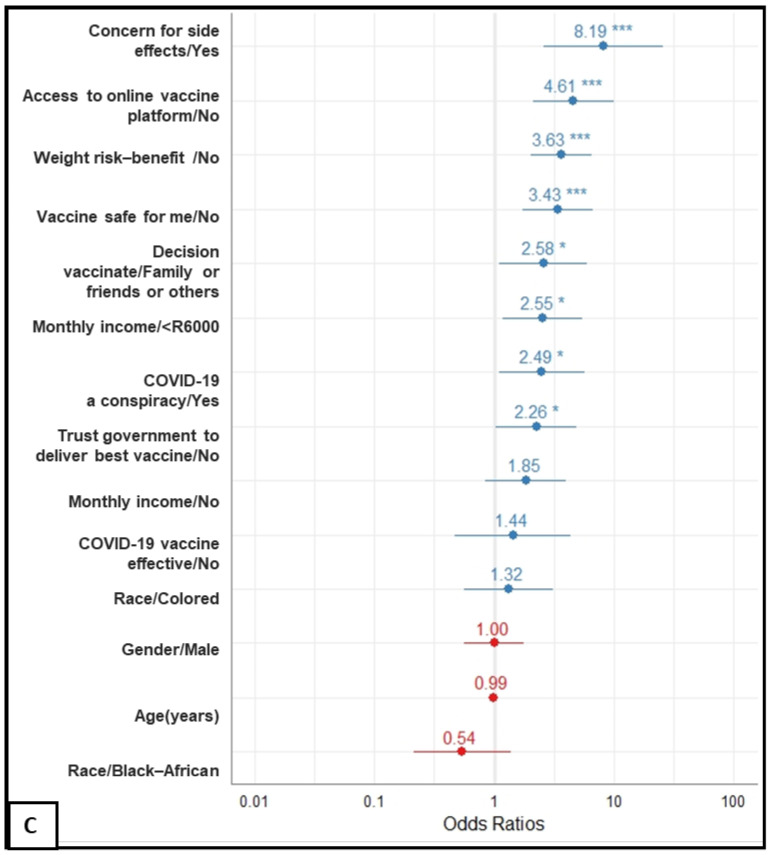
Forest plots displaying factors associated with vaccine hesitance among respondents from four local communities in South Africa, from 6 June to 30 June 2021. Plots (**A**–**C**) indicate models adjusted for sociodemographic factors, COVID-19 related factors and combination of (**A**,**B**), respectively. Of note, all models are adjusted for gender (binary) and for age (continuous). The vertical line, slightly thicker than the other grid lines (x-axis position 1) indicates the neutral line, i.e., the vertical intercept that indicates no effect. Points in the plots and related horizontal lines represent the estimated odds ratio and their corresponding 95% confidence interval (CI) of COVID-19 vaccine hesitancy defined as uncertainty or intention to refuse COVID-19 vaccination, respectively. Blue and red colors indicate odds of hesitancy and of acceptance respectively. Asterisks indicate the significance level of the *p*-values (*: *p* < 0.05, **: *p* < 0.01, ***: *p* < 0.001). Exact 95% CI and *p*-values are provided in Table 2.

Figure 2A–C displays estimates of factors independently associated with COVID-19 vaccine hesitancy, and Table 2 shows both bivariate and multivariable logistic regression details, including 95% confidence intervals and exact *p*-values.

Firstly, after adjusting for differences in respondent sociodemographic characteristics, in a monotonic pattern, people aged between 35–44, 45–54 and 55+ years exhibited 54% (aOR, 0.46; 95% CI, 0.31 to 0.68), 63% (aOR, 0.37; 95% CI, 0.24 to 0.56) and 70% (aOR, 0.30; 95% CI, 0.19 to 0.46) reduction in the odds of delaying or refusing the COVID-19 vaccine once available, respectively, compared to those aged between 18–24 years. Compared to Indian/Asian respondents, Colored respondents had a nearly 4-fold higher odds (aOR, 3.68; 95% CI, 2.18 to 6.46) of not intending to be vaccinated against COVID-19 infection. Although Black respondents were almost 2.5 times more likely of being hesitant in the bivariate model as compared to Indian/Asian respondents, the statistical significance did not persist after adjusting for other sociodemographic factors in the model. There were notable socioeconomic differences in COVID-19 vaccine uptake among participants with those earning below R6000/month (aOR, 1.79; 95% CI, 1.17 to 2.76) or nothing at all per month (aOR, 1.84; 95% CI, 1.12 to 3.07) showing a significant increase in odds of being reluctant to vaccinate for COVID-19 compared to those who reported a monthly income of R6000 or above. No statistical difference was observed between males and females.

Secondly, in the model adjusting for known behavioral and social drivers of general immunization program and adapted for the COVID-19 pandemic, independent factors associated with COVID-19 vaccine hesitancy were lack of access to the government COVID-19 online platform (aOR, 4.75; 95% CI, 2.15 to 10.37), not weighing risk–benefit before deciding to vaccinate (aOR, 3.46; 95% CI, 1.90 to 6.33), concern about safety for oneself (aOR, 2.89; 95% CI, 1.46 to 5.70), concern about side effects (aOR, 11.41; 95% CI, 3.5 to 50.80), distrust in the government’s capability to deliver the best vaccine (aOR, 3.0; 95% CI, 1.33 to 6.77), belief in conspiracy theories with regards to the origin of SARS-CoV-2 (aOR, 3.01; 95% CI, 1.32 to 6.77), and not holding oneself the final decision to vaccinate (aOR, 2.47; 95% CI, 1.06 to 5.77).

Lastly, by adjusting for factors statistically significant in models 1 and 2 while controlling for both age (as continuous variables) and gender, we found that all known behavioral and social drivers were slightly reduced (except the odds for concern about safety that increased: aOR, 2.89 vs. 3.09) but remained independently associated with the high likelihood of delaying or refusing the COVID-19 vaccine once available. Considering sociodemographic factors, having a monthly income below R6000 was the most significant factor remaining independently associated with the unwillingness to vaccinate for COVID-19 once available.

## 4. Discussion

In this community-based cross-sectional study, one-third of participants reported their intention to delay or refuse the vaccine once available. Factors independently associated with COVID-19 vaccine hesitancy covered various aspects of the BeSD domains, including sociodemographic variables (age, race, low monthly income), practical issues (lack of access to government COVID-19 registration platform), thinking and feeling (government distrust, concern about safety/side effect, misinformation) and social processes (not holding oneself responsible for the final decision to vaccinate). Our findings point to three features that should shape an approach to vaccine promotion in South Africa, which may be applicable to other LMIC contexts.

### 4.1. Community Profiles: Sociodemographic Variables and Trust in Government

Regardless of education level, the likelihood of vaccine hesitancy was predicted by young age [31,32], racial disparity [33,34], and low monthly income [35,36]. Consistent with other COVID-19 studies [37,38], we also found government distrust was associated with vaccine hesitancy among our respondents. Since people with low socioeconomic status have been unequally impacted, this might explain in part the association we found between vaccine hesitancy, distrust, low monthly income, and ethnic disparity [39,40,41]. Vaccination uptake in Africa is associated with the level of trust communities have for government. For example, one study of child vaccination rates in Africa found a significantly reduced rate of vaccination in regions where the local population was distrustful of local authorities, with a one standard deviation increase in the institutional mistrust index being associated with a 10% increase in the likelihood that a child had missed any of the eight basic vaccines [42].

### 4.2. Priority Messaging: Addressing Concerns about Vaccine Safety and Side Effects

Our findings align with earlier studies that found vaccine hesitancy among adults (parents/caregivers) and is associated with concerns about side effects [19,27]. For example, a range of studies conducted before [43,44,45] and after [46,47] the introduction of the school-based human papilloma virus (HPV) vaccination program in 2014 all revealed that parents might have various concerns regarding the HPV vaccine, including its safety and efficacy, and its short- and long-term side effects. A global multi-country study analyzing COVID-19 vaccine willingness in June 2020 [14] with a one-year follow-up in June 2021 [48] showed that the level of hesitancy remained high in LMICs and urged health policymakers to provide accurate information regarding efficacy and safety of COVID-19 vaccines in comparison to illness risk. The Ipsos [49] study (country = 15, participants = 5932) conducted in partnership with the World Economic Forum found that perceptions of risk to health (side effects and speed of vaccine to market) perfectly described the reason for unwillingness to vaccinate against COVID-19. A similar pattern was reported in Ghana [50] and Nigeria [51]. Furthermore, in a survey of 5416 Africans, 37% of participants were hesitant to accept the COVID-19 immunization once available to them (with the highest level of hesitancy observed among participants from Central Africa; 67%), and 79% were concerned about its negative effects [52]. Increasingly in South Africa, more people are engaging with fake information about vaccination which informs a false picture of risk [47]. We found not having access to government information platforms and believing in conspiracy theories strongly predicted hesitancy towards the COVID-19 vaccine. Where social media has been allowed to dominate vaccine messaging, one global study found that this directly impacted vaccine uptake by increasing public doubts of vaccine safety [53].

### 4.3. The Value of Socially Embedded Approaches to Vaccine Promotion

Approaches to vaccine hesitancy should also consider addressing personal motivation and social influences as well as creating an enabling environment [22,54,55]. Most participants in our study believed that people who do not get the vaccine are taking risks both for their own health and that of the wider community. Additionally, respondents expressed optimism that the majority of adults in their local communities will receive a COVID-19 vaccination and that a variety of stakeholders, including family, friends, and local and political leaders, will advocate for them to do so. This is critical to behavior change in a community since personal, interpersonal, community and social/political factors need to be addressed for a change in behavior to occur in many people and then be maintained and sustained [56]. This is because the enablers or barriers to behavior change can be readily identified. For example, if a local faith leader is speaking out against vaccination it will most likely have an impact on the social norms of the local community. Furthermore, we found that half of the participants considered healthcare workers (nurse/doctor) as their reliable source of information [15] when confronted with confusing information, while more than half of respondents indicated that they would prefer to be vaccinated in a health-related facility such as a hospital, clinic, or pharmacy. Consequently, if vaccine hesitancy is high among healthcare workers [57] or if the local clinic has a reputation for providing a poor service, then these too will affect vaccine uptake as they will constitute a barrier to an enabling environment.

### 4.4. Study Limitations

Our findings should be interpreted considering the following limitations. Firstly, we used the WHO BeSD instrument adjusted for COVID-19 that does not include type of vaccine and associated efficacy, whilst current evidence suggests that hesitancy towards COVID-19 vaccines is associated with both the vaccine platform and vaccine efficacy [58,59]. Secondly, at the time of our survey, vaccines were not available for all age groups and in all communities. Consequently, respondents’ actual vaccine uptake might be influenced by factors such as access and availability of vaccines. Similarly, other events that might have an influence on vaccine uptake, such as suspending one vaccine candidate while confirming serious adverse events following immunization, have occurred during and after our study. Future studies should consider a longitudinal design to better understand the highly fluctuating landscape to characterize change in the vaccine uptake continuum over time. Thirdly, although most of our participants were surveyed using a face-to-face interview, we also utilized web-based methods to reach respondents in wards during hard lockdowns. By using an e-survey, at high risk of vaccine hesitancy participants (low monthly income, minority groups, inmates, undocumented migrants) could have been missed and therefore bias the truth towards the null. Lastly, these findings cannot be extrapolated to the national population or other communities due to the use of convenience sampling, as well as the differences that may exist between communities. Despite these limitations, our main findings strikingly align with those from other studies in South Africa, as well as those from global surveys and metanalyses. Furthermore, we took care to include respondents from urban, urban-informal, and semi-rural communities to minimize social disparities and to analyze a significant number of predictors known to impact vaccine willingness by using the BeSD tool. Our findings also contribute data on the use of the BeSD model in the global south.

## 5. Conclusions

Our research examines factors related to vaccination hesitancy at the community level in South Africa using the World Health Organization Behavioral Social Drivers of COVID-19 vaccination tool, which seems to be effective in the global south. The most effective interventions against vaccination hesitancy are those that are based on scientific facts and situational analysis. We found that addressing trust issues is critical to improving vaccine uptake. Health systems and government agencies should collaborate to build trust in these local communities by utilizing peer-to-peer and credible messenger strategies. This should include interacting with each individual segment of the vaccine-hesitant population while taking into account the type and severity of their reluctance. Furthermore, since one-way health messages cannot accomplish adequate behavior change, an organizational health literacy process with nuanced vaccination messaging tailored to community issues such as economic and racial disparities is necessary to sustain the COVID-19 vaccine program’s implementation. This public engagement strategy should look at not just tailored information, but also effective approaches for reaching out to those who identify as outright refusers.

## Figures and Tables

**Table 1:** Sociodemographic characteristics of 1193 South Africans included in the VaxScenes Study from the 6 June to the 30 July 2021.

	All	Acceptance	Hesitancy	*p*-Values
	n = 1193	n = 811 (68%)	n = 382 (32%)	
**Age** (Years)/Mean(SD)	39.4 (14.7)	41.3 (14.9)	35.4 (13.5)	<0.001
**Age Groups:**				<0.001
18–24 Years	203 (17.7%)	109 (13.9%)	94 (25.8%)	
25–34 Years	294 (25.6%)	184 (23.4%)	110 (30.1%)	
35–44 Years	253 (22.0%)	181 (23.1%)	72 (19.7%)	
45–54 Years	200 (17.4%)	152 (19.4%)	48 (13.2%)	
> 55 Years	200 (17.4%)	159 (20.3%)	41 (11.2%)	
**Gender (all):**				0.225
Female	636 (53.4%)	442 (54.6%)	194 (50.8%)	
Male	543 (45.6%)	361 (44.6%)	182 (47.6%)	
Other	12 (1.01%)	6 (0.74%)	6 (1.57%)	
**Race:**				<0.001
Indian/Asian	179 (15.6%)	154 (19.7%)	25 (6.78%)	
Black	573 (49.9%)	408 (52.3%)	165 (44.7%)	
Colored	397 (34.6%)	218 (27.9%)	179 (48.5%)	
**Education:**				0.942
Matric/tertiary	769 (64.7%)	523 (64.8%)	246 (64.4%)	
Not/Some schooling	420 (35.3%)	284 (35.2%)	136 (35.6%)	
**Employment:**				0.001
Employed	582 (48.9%)	423 (52.4%)	159 (41.6%)	
Unemployed	608 (51.1%)	385 (47.6%)	223 (58.4%)	
**Income:**				<0.001
≥R6000/month	372 (31.4%)	288 (35.6%)	84 (22.2%)	
<R6000/month	449 (37.9%)	292 (36.1%)	157 (41.5%)	
None	365 (30.8%)	228 (28.2%)	137 (36.2%)	
**Self-reported comorbidity**	254 (23.2%)	183 (24.5%)	71 (20.3%)	0.153

Data are expressed as number (percentage) unless otherwise specified. R = rand, the South African currency. R6000, about 415 American dollars. SD: standard deviation.

**Table 2:** Determinants associated with COVID-19 vaccine hesitancy among South Africans surveyed from the 6th of June to the 30th of July 2021. Multivariate logistic models consider first sociodemographic factors, then COVID-19 and COVID-19 vaccine-related factors, then the two together.

		Vaccine Uptake Willingness n = 1193		Bivariate Model	Multivariable Models		
					Model 1	Model 2	Model 3
Variables		Acceptance	Hesitancy	cOR (95% CI, *p*-Values)	aOR (95% CI, *p*-Values)	aOR (95% CI, *p*-Values)	aOR (95% CI, *p*-Values)
**Age groups**	18–24 Years	109 (53.7)	94 (46.3)	**Reference**	**Reference**	_	_
	25–34 Years	184 (62.6)	110 (37.4)	0.69 (0.48–1.00, *p* = 0.048)	0.78 (0.51–1.18, *p* = 0.238)	_	_
	35–44 Years	181 (71.5)	72 (28.5)	0.46 (0.31–0.68, *p* < 0.001)	0.52 (0.33–0.82, *p* = 0.005)	_	_
	45–54 Years	152 (76.0)	48 (24.0)	0.37 (0.24–0.56, *p* < 0.001)	0.39 (0.24–0.63, *p* < 0.001)	_	_
	>55 Years	159 (79.5)	41 (20.5)	0.30 (0.19–0.46, *p* < 0.001)	0.32 (0.18–0.54, *p* < 0.001)	_	_
**Gender**	Female	442 (69.5)	194 (30.5)	**Reference**	**Reference**	_	_
	Male	361 (66.5)	182 (33.5)	1.15 (0.90–1.47, *p* = 0.268)	0.92 (0.69–1.22, *p* = 0.565)	_	_
**Race**	Indian/Asian	154 (86.0)	25 (14.0)	**Reference**	**Reference**	**_**	**Reference**
	Black	408 (71.2)	165 (28.8)	2.49 (1.60–4.02, *p* < 0.001)	1.45 (0.83–2.62, *p* = 0.206)	_	0.60 (0.23–1.56, *p* = 0.291)
	Colored	218 (54.9)	179 (45.1)	5.06 (3.22–8.22, *p* < 0.001)	3.68 (2.18–6.46, *p* < 0.001)	_	1.48 (0.62–3.62, *p* = 0.378)
**Education**	Matric/tertiary	523 (68.0)	246 (32.0)	**Reference**	**Reference**	_	_
	Not/some schooling	284 (67.6)	136 (32.4)	1.02 (0.79–1.31, *p* = 0.890)	1.15 (0.83–1.60, *p* = 0.391)	_	_
**Employment**	Employed	423 (72.7)	159 (27.3)	**Reference**	**Reference**	_	_
	Unemployed	385 (63.3)	223 (36.7)	1.54 (1.21–1.97, *p* = 0.001)	0.96 (0.65-1.40, *p* = 0.819)	_	_
**Income**	≥R6000/month	288 (77.4)	84 (22.6)	**Reference**	**Reference**	**_**	**Reference**
	<R6000/month	292 (65.0)	157 (35.0)	1.84 (1.35–2.52, *p* < 0.001)	1.79 (1.17-2.76, *p* = 0.008)	_	2.53 (1.18–5.50, *p* = 0.018)
	None	228 (62.5)	137 (37.5)	2.06 (1.50–2.85, *p* < 0.001)	1.84 (1.12-3.07, *p* = 0.017)	_	1.78 (0.80–3.95, *p* = 0.155)
**At least one** **comorbidity**	No	565 (67.0)	278 (33.0)	**Reference**	**Reference**	**_**	**Reference**
	Yes	183 (72.0)	71 (28.0)	0.79 (0.58–1.07, *p* = 0.132)	1.01 (0.71-1.43, *p* = 0.963)	_	0.99 (0.47–2.02, *p* = 0.978)
**Previous history** **of vaccination**	Yes	750 (69.3)	333 (30.7)	**Reference**	**_**	**Reference**	_
	No	61 (56.0)	48 (44.0)	1.77 (1.18–2.64, *p* = 0.005)	_	1.49 (0.35–6.29, *p* = 0.583)	_
**Receive social** **grant**	Yes	567 (69.1)	253 (30.9)	**Reference**	**_**	**Reference**	_
	No	241 (65.5)	127 (34.5)	1.18 (0.91–1.53, *p* = 0.212)	_	1.34 (0.70–2.53, *p* = 0.371)	_
**Have lost** **income**	Yes	594 (70.1)	253 (29.9)	**Reference**	**_**	**Reference**	_
	No	217 (62.7)	129 (37.3)	1.40 (1.07–1.81, *p* = 0.013)	_	1.45 (0.75–2.77, *p* = 0.262)	_
**Know people** **who lost income**	Yes	301 (72.2)	116 (27.8)	**Reference**	**_**	**Reference**	_
	No	510 (65.7)	266 (34.3)	1.35 (1.04–1.76, *p* = 0.023)	_	0.91 (0.46–1.84, *p* = 0.791)	_
**Impacted by** **COVID-19 death**	Yes	279 (65.8)	145 (34.2)	**Reference**	**_**	**Reference**	_
	No	532 (69.2)	237 (30.8)	0.86 (0.67–1.10, *p* = 0.231)	_	0.69 (0.35–1.37, *p* = 0.284)	_
**Know people** **who lost lives**	Yes	255 (72.2)	98 (27.8)	**Reference**	**_**	**Reference**	_
	No	556 (66.2)	284 (33.8)	1.33 (1.01–1.75, *p* = 0.041)	_	1.13 (0.55–2.36, *p* = 0.749)	_
**Will pay for** **COVID-19 vaccine**	Yes	73 (50.3)	72 (49.7)	**Reference**	**_**	**Reference**	_
	No	732 (70.4)	308 (29.6)	0.43 (0.30–0.61, *p* < 0.001)	_	1.16 (0.53–2.67, *p* = 0.713)	_
**Can access** **COVID-19 online** **platform**	Yes	182 (92.4)	15 (7.6)	**Reference**	**_**	**Reference**	**Reference**
	No	223 (68.0)	105 (32.0)	5.71 (3.31–10.53, *p* < 0.001)	_	4.57 (2.15–10.37, *p* < 0.001)	4.22 (2.00–9.52, *p* < 0.001)
**Perceive risk** **of being infected**	Yes	767 (70.5)	321 (29.5)	**Reference**	**_**	**Reference**	_
	No	40 (40.4)	59 (59.6)	3.52 (2.32–5.41, *p* < 0.001)	_	1.46 (0.40–5.01, *p* = 0.557)	_
**Weigh risk–benefit**	Yes	537 (85.4)	92 (14.6)	**Reference**	**_**	**Reference**	**Reference**
	No	273 (48.5)	290 (51.5)	6.20 (4.72–8.20, *p* < 0.001)	_	3.46 (1.90–6.33, *p* < 0.001)	3.66 (2.02–6.69, *p* < 0.001)
**Vaccine not** **safe for me**	Yes	693 (83.0)	142 (17.0)	**Reference**	**_**	**Reference**	**Reference**
	No	115 (32.5)	239 (67.5)	10.14 (7.64–13.55, *p* < 0.001)	_	2.89 (1.46–5.70, *p* = 0.002)	3.09 (1.56–6.15, *p* = 0.001)
**Concern about** **side effects**	Yes	241 (76.8)	73 (23.2)	**Reference**	**_**	**Reference**	**Reference**
	No	569 (64.8)	309 (35.2)	1.79 (1.34–2.42, *p* < 0.001)	_	11.41 (3.50–50.80, *p* < 0.001)	7.47 (2.63–26.49, *p* = 0.001)
**Vaccine not** **effective**	Yes	772 (74.1)	270 (25.9)	**Reference**	**_**	**Reference**	**Reference**
	No	39 (25.8)	112 (74.2)	8.21 (5.61–12.25, *p* < 0.001)	_	1.25 (0.41–3.91, *p* = 0.693)	1.40 (0.46–4.36, *p* = 0.558)
**Trust HCW for** **information**	Yes	729 (73.0)	269 (27.0)	**Reference**	**_**	**Reference**	_
	No	77 (40.7)	112 (59.3)	3.94 (2.86–5.45, *p* < 0.001)	_	0.76 (0.30–1.84, *p* = 0.548)	_
**Trust government** **for best vaccine**	Yes	711 (77.2)	210 (22.8)	**Reference**	**_**	**Reference**	**Reference**
	No	92 (35.2)	169 (64.8)	6.22 (4.63–8.40, *p* < 0.001)	_	3.00 (1.33–6.72, *p* = 0.007)	2.23 (1.01–4.89, *p* = 0.045)
**COVID-19 not a** **conspiracy**	Yes	666 (81.2)	154 (18.8)	**Reference**	**_**	**Reference**	**Reference**
	No	145 (38.9)	228 (61.1)	6.80 (5.19–8.95, *p* < 0.001)	_	3.01 (1.32–6.77, *p* = 0.008)	2.55 (1.11–5.82, *p* = 0.027)
**Will decide myself** **for vaccination**	Yes	704 (75.2)	232 (24.8)	**Reference**	**_**	**Reference**	**Reference**
	No	87 (37.8)	143 (62.2)	4.99 (3.69–6.79, *p* < 0.001)	_	2.47 (1.06–5.77, *p* = 0.036)	2.42 (1.01–5.75, *p* = 0.045)
**Will vaccinate** **to protect others**	Yes	690 (79.0)	183 (21.0)	**Reference**	**_**	**Reference**	_
	No	119 (37.4)	199 (62.6)	6.31 (4.78–8.36, *p* < 0.001)	_	1.67 (0.81–3.40, *p* = 0.161)	_

Data are expressed as number (percentage). Model 1: Model adjusts for sociodemographic factors associated with vaccine hesitancy. Model 2: Model adjusts for COVID-19 and COVID-19 vaccine-related factors as well as for age as continuous and gender as a binary variable. Model 3: Model considers factors statistically significant in both model 1 and 2 while holding constant age as continuous variable. Abbreviations: cOR: crude odds ratio, aOR: adjusted odds ratio, CI: confidence interval, HCW: health care worker, R = rand, South African currency, R600 is approximately about 415 American dollars. Participants earning less than R6000 per month were classified as having a low monthly income, while those earning more than R6000 per month were classified as having a moderate to high monthly income.

## Data Availability

The datasets used and/or analyzed during the current study are available from the corresponding author upon a reasonable request.

## Supplementary Materials

The following supporting information can be downloaded at: https://www.mdpi.com/article/10.3390/vaccines10030353/s1. Table S1: Four case study sites by province, ward, settlement type, population, and COVID-19 statistics as of 29 January 2021; Table S2: Supplemental questions related to COVID-19 and COVID-19 vaccine; Figure S1: Age groups distribution of COVID-19 vaccine uptake. Analyses by gender (A), level of education (B) and study sites (C) among respondents from four local communities in South Africa, from 6 June to 30 June 2021.
