# Supplementary material for: Predictors of COVID-19 Vaccine Hesitancy in South African Local Communities: The VaxScenes Study
Source: Vaccines (Basel). 2022 Feb 25;10(3):353. doi: 10.3390/vaccines10030353 (PMC8951818; doi:10.3390/vaccines10030353)
Supplement: Supplementary file 1 [file vaccines-10-00353-s001.zip › vaccines-1580855-supplementary.pdf]

Predictors of COVID-19 Vaccine Hesitancy in South African Local Communities: The VaxScenes Study

Authors

Patrick DMC Katoto[1,2,3], Saahier Parker[4], Nancy Coulson[5], Nirvana Pillay[5], Sara Cooper[1], Anelisa Jaca[6], Edison Mavundza[1], Gregory Houston[4], Candice Groenewald[4], Zaynab Essack[4], Jane Simmonds[6], Londiwe Deborah Shandu[1], Marilyn Couch[4], Nonkululeko Khuzwayo[4], Nobukhosi Ncube[5], Phelele Bhengu[1,7], Heidi van Rooyen[4,8], *Charles Shey Wiysonge[1,9,10]

Supplementary materials

Table S1: Four case study sites by province, ward, settlement type, population, and COVID-19 statistics as of 29 January 2021.

Case Study Site	Province	Ward	Type	Population	Provincial COVID-19 Data*
Alexandra	Gauteng	Ward 76, Region E City of Johannesburg	Urban, informal	798 014 (2011 Census)	386 770 cases<br>10 195 deaths
Rylands Estate	Western Cape	Ward 46, Athlone, City of Cape Town	Urban	32 199	268 507 cases<br>10,247 deaths
Sweetwaters	KwaZulu Natal	Vulundlela, Umgungundlovu District Municipality	Semi-Rural	18 587	311 877 cases<br>8 443deaths
Wentworth	KwaZulu Natal	Ward 66, eThekwini Municipality	Urban	145 949 (2016 Census)	

*Source:https://sacoronavirus.co.za/2021/01/29/update-on-COVID-19-29th-january-2021/.

Table S2: Supplemental questions related to COVID-19 and COVID-19 vaccine.

Variables	Respondents n (%)
**Do you think the following people in your community would want you to get a COVID-19 vaccine?**	
**Family and friend**	
Yes	701 (59.7%)
No	176 (15.0%)
Not sure	297 (25.3%)
**The local ward councillor/s**	
Yes	714 (60.9%)
No	88 (7.50%)
Not sure	371 (31.6%)
**Local leaders in political parties**	
Yes	692 (60.1%)
No	95 (8.25%)
Not sure	365 (31.7%)
**Traditional leaders**	
Yes	371 (32.4%)
No	186 (16.2%)

Not sure	589 (51.4%)
**Religious leaders**	
Yes	660 (57.3%)
No	146 (12.7%)
Not sure	346 (30.0%)
**Do you think most adults you know in this local community will get a COVID-19 vaccine, if government makes it available to them?**	
Yes	855 (73.1%)
No	84 (7.19%)
Not sure	230 (19.7%)
**What do you think would help ensure that as many people as possible in your community get the COVID-19 vaccine?**	
Make vaccines available as quickly as possible to all adults	880 (73.8%)
Ensure the COVID-19 vaccine is free	885 (74.2%)
Publicly vaccinate community leaders	726 (60.9%)
Provide more education about the COVID-19 vaccine	827 (69.3%)
Involve religious and community leaders in COVID-19 education programmes	800 (67.1%)
Encourage community participation through ward councilors	765 (64.1%)
Make information easily available online	704 (59.0%)
**If you hear confusing information related to the COVID-19 vaccine, who would you go to for reliable information?**	
A family member	154 (13.7%)

A friend or neighbor	29 (2.57%)
A nurse or doctor	594 (52.7%)
A community outreach worker (community health worker)	60 (5.32%)
A traditional healer	6 (0.53%)
An online search	238 (21.1%)
I don’t check information that I receive	47 (4.17%)
**Where would you prefer to get a COVID-19 vaccine?**	
Hospital	222 (19.6%)
Health clinic	298 (26.3%)
Workplace	58 (5.12%)
Pharmacy	206 (18.2%)
Community centre, meeting hall, or local shopping centre	205 (18.1%)
I don’t want the vaccine	144 (12.7%)
**Do you think people who do not get the COVID-19 vaccine are taking a risk with**	
Their own health	159 (13.6%)
The health of the wider community	68 (5.80%)
Both their own health AND that of the wider community	775 (66.1%)
They are not taking unnecessary risks	170 (14.5%)
**How much do you trust the National Government to manage the rollout of the COVID-19 vaccine for South Africa?**	

Not at all	254 (21.3%)
A little	274 (23.0%)
Moderately	396 (33.2%)
Very much	267 (22.4%)
**How much do you trust the pharmaceutical industry in its development of the COVID-19 vaccine?**	
Not at all	198 (16.6%)
A little	249 (20.9%)
Moderately	439 (36.9%)
Very much	305 (25.6%)
**How much trust do you have in the scientists advising National Government about COVID-19 vaccine?**	
Not at all	183 (15.4%)
A little	206 (17.3%)
Moderately	396 (33.3%)
Very much	405 (34.0%)
**Will you continue to wear a mask after you are vaccinated?**	
Yes	773 (94.6%)
No	28 (3.43%)
Not sure	16 (1.96%)

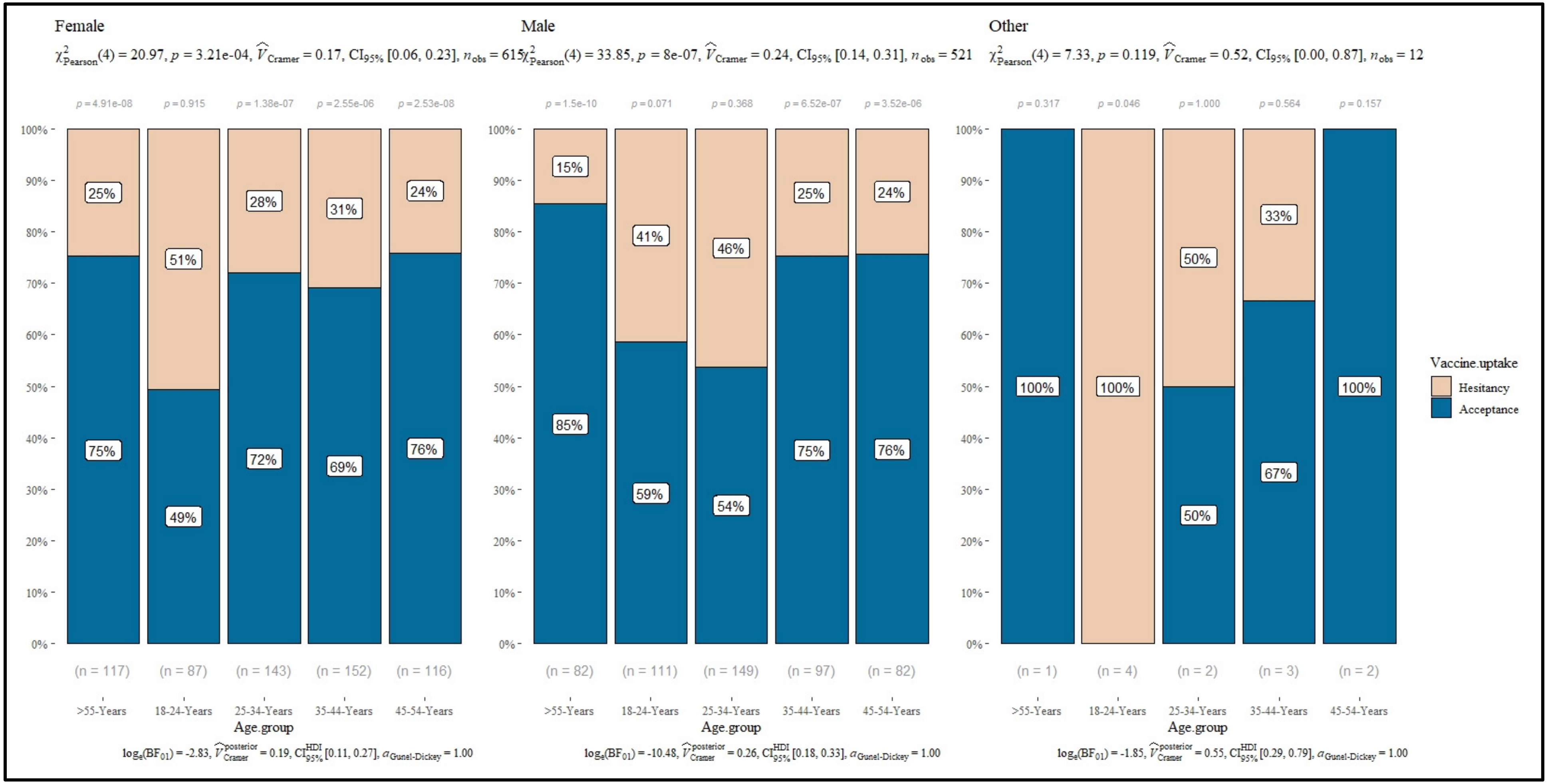

Female
Male
Other
p = 4.91e-08
p = 0.915
p = 1.38e-07
p = 2.55e-06
p = 2.53e-08
p = 1.5e-10
p = 0.071
p = 0.368
p = 6.52e-07
p = 3.52e-06
p = 0.317
p = 0.046
p = 1.000
p = 0.564
p = 0.157
(n = 117) (n = 87) (n = 143) (n = 152) (n = 116)
(n = 82) (n = 111) (n = 149) (n = 97) (n = 82)
(n = 1) (n = 4) (n = 2) (n = 3) (n = 2)
>55-Years 18-24-Years 25-34-Years 35-44-Years 45-54-Years
Age.group
Vaccine.uptake
Hesitancy
Acceptance

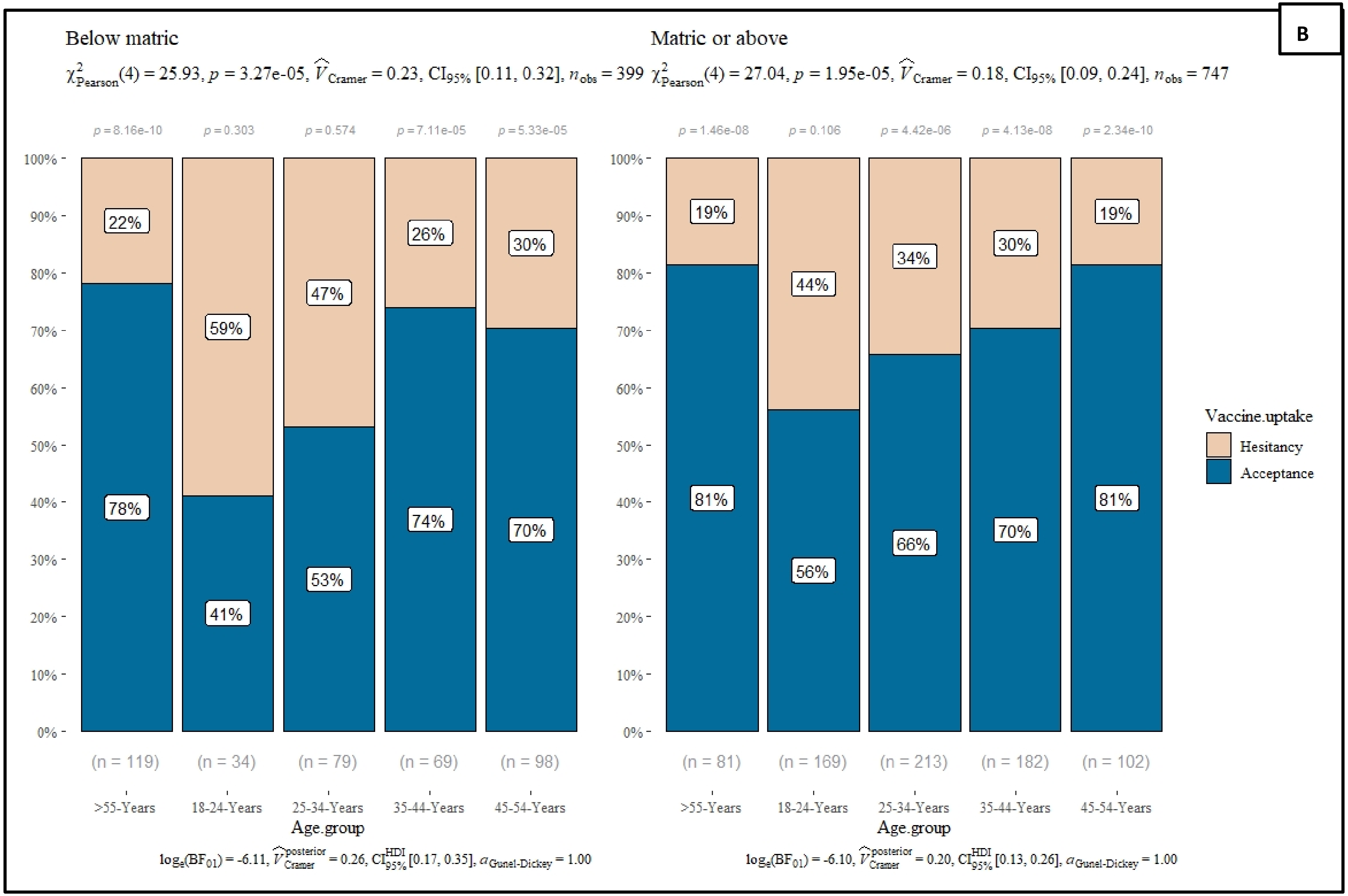
B
Below matric
$\chi^2_{Pearson}(4) = 25.93$, $p = 3.27e-05$, $\widehat{V}_{Cramer} = 0.23$, $CI_{95\%}$ [0.11, 0.32], $n_{obs} = 399$
Matric or above
$\chi^2_{Pearson}(4) = 27.04$, $p = 1.95e-05$, $\widehat{V}_{Cramer} = 0.18$, $CI_{95\%}$ [0.09, 0.24], $n_{obs} = 747$
p = 8.16e-10
p = 0.303
p = 0.574
p = 7.11e-05
p = 5.33e-05
p = 1.46e-08
p = 0.106
p = 4.42e-06
p = 4.13e-08
p = 2.34e-10
22%
78%
59%
41%
47%
53%
26%
74%
30%
70%
19%
81%
44%
56%
34%
66%
30%
70%
19%
81%
(n = 119)
(n = 34)
(n = 79)
(n = 69)
(n = 98)
(n = 81)
(n = 169)
(n = 213)
(n = 182)
(n = 102)
>55-Years
18-24-Years
25-34-Years
35-44-Years
45-54-Years
Age.group
$\log_e(BF_{01}) = -6.11$, $\widehat{V}^{posterior}_{Cramer} = 0.26$, $CI^{HDI}_{95\%}$ [0.17, 0.35], $a_{Gunel-Dickey} = 1.00$
$\log_e(BF_{01}) = -6.10$, $\widehat{V}^{posterior}_{Cramer} = 0.20$, $CI^{HDI}_{95\%}$ [0.13, 0.26], $a_{Gunel-Dickey} = 1.00$
Vaccine.uptake
Hesitancy
Acceptance

C

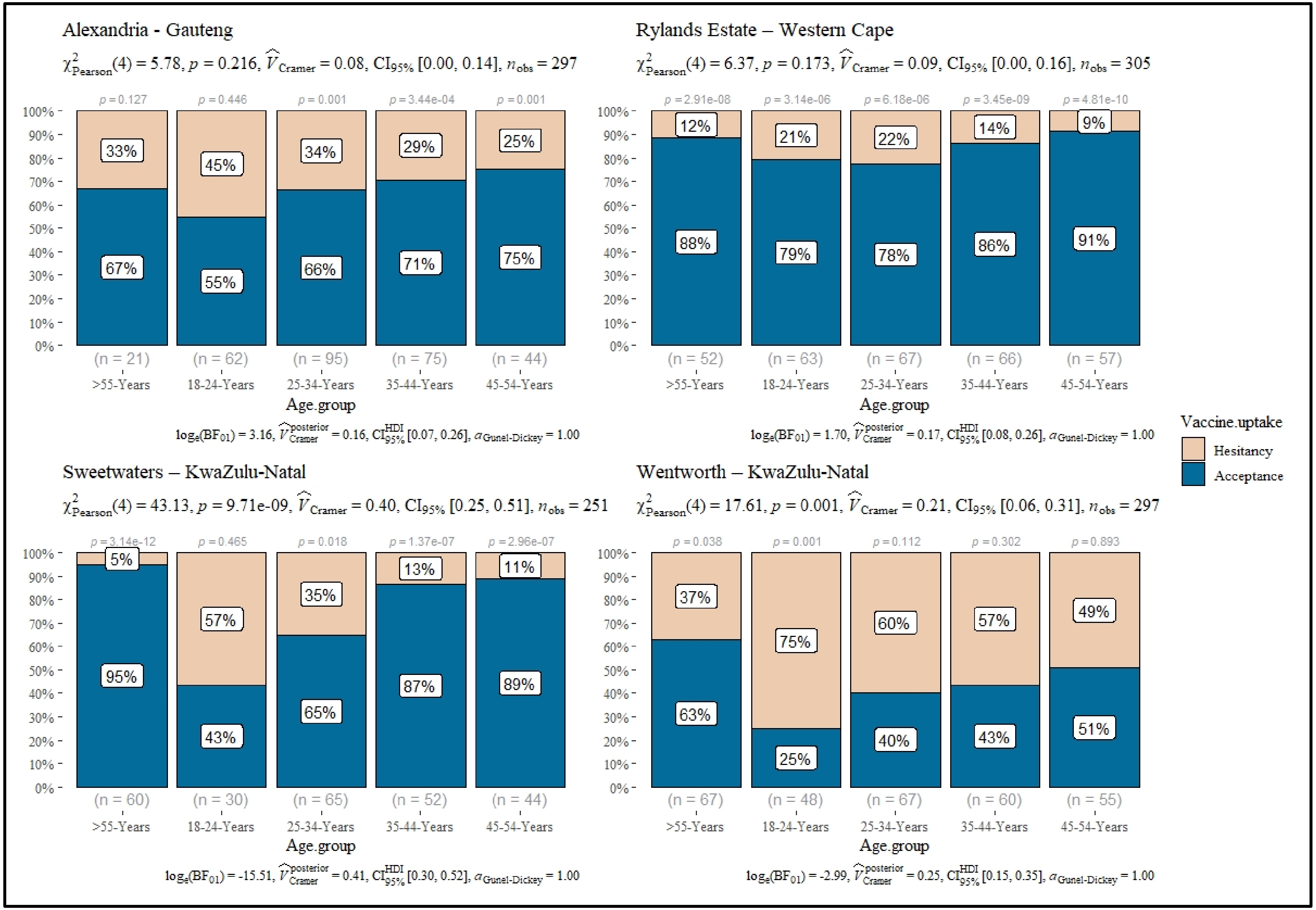

Alexandria - Gauteng
$\chi^2_{Pearson}(4) = 5.78$, $p = 0.216$, $\widehat{V}_{Cramer} = 0.08$, $CI_{95\%}$ [0.00, 0.14], $n_{obs} = 297$
p = 0.127
p = 0.446
p = 0.001
p = 3.44e-04
p = 0.001
33%
45%
34%
29%
25%
67%
55%
66%
71%
75%
(n = 21)
(n = 62)
(n = 95)
(n = 75)
(n = 44)
>55-Years
18-24-Years
25-34-Years
35-44-Years
45-54-Years
Age.group
$\log_e(BF_{01}) = 3.16$, $\widehat{V}^{posterior}_{Cramer} = 0.16$, $CI^{HDI}_{95\%}$ [0.07, 0.26], $a_{Gunel-Dickey} = 1.00$
Rylands Estate – Western Cape
$\chi^2_{Pearson}(4) = 6.37$, $p = 0.173$, $\widehat{V}_{Cramer} = 0.09$, $CI_{95\%}$ [0.00, 0.16], $n_{obs} = 305$
p = 2.91e-08
p = 3.14e-06
p = 6.18e-06
p = 3.45e-09
p = 4.81e-10
12%
21%
22%
14%
9%
88%
79%
78%
86%
91%
(n = 52)
(n = 63)
(n = 67)
(n = 66)
(n = 57)
>55-Years
18-24-Years
25-34-Years
35-44-Years
45-54-Years
Age.group
$\log_e(BF_{01}) = 1.70$, $\widehat{V}^{posterior}_{Cramer} = 0.17$, $CI^{HDI}_{95\%}$ [0.08, 0.26], $a_{Gunel-Dickey} = 1.00$
Vaccine.uptake
Hesitancy
Acceptance
Sweetwaters – KwaZulu-Natal
$\chi^2_{Pearson}(4) = 43.13$, $p = 9.71e-09$, $\widehat{V}_{Cramer} = 0.40$, $CI_{95\%}$ [0.25, 0.51], $n_{obs} = 251$
p = 3.14e-12
p = 0.465
p = 0.018
p = 1.37e-07
p = 2.96e-07
5%
57%
35%
13%
11%
95%
43%
65%
87%
89%
(n = 60)
(n = 30)
(n = 65)
(n = 52)
(n = 44)
>55-Years
18-24-Years
25-34-Years
35-44-Years
45-54-Years
Age.group
$\log_e(BF_{01}) = -15.51$, $\widehat{V}^{posterior}_{Cramer} = 0.41$, $CI^{HDI}_{95\%}$ [0.30, 0.52], $a_{Gunel-Dickey} = 1.00$
Wentworth – KwaZulu-Natal
$\chi^2_{Pearson}(4) = 17.61$, $p = 0.001$, $\widehat{V}_{Cramer} = 0.21$, $CI_{95\%}$ [0.06, 0.31], $n_{obs} = 297$
p = 0.038
p = 0.001
p = 0.112
p = 0.302
p = 0.893
37%
75%
60%
57%
49%
63%
25%
40%
43%
51%
(n = 67)
(n = 48)
(n = 67)
(n = 60)
(n = 55)
>55-Years
18-24-Years
25-34-Years
35-44-Years
45-54-Years
Age.group
$\log_e(BF_{01}) = -2.99$, $\widehat{V}^{posterior}_{Cramer} = 0.25$, $CI^{HDI}_{95\%}$ [0.15, 0.35], $a_{Gunel-Dickey} = 1.00$

Figure S1: **Age-groups distribution of COVID-19 vaccine uptake.** Analyses by gender (A), level of education (B) and study sites (C) among respondents from four local communities in South Africa, from 6 June to 30 June 2021.
